# Genotypic Variation in Root System Architecture Is Associated with Total Plant Nitrogen Accumulation in Peanut (*Arachis hypogaea* L.) Under Low-Nitrogen Conditions

**DOI:** 10.3390/plants15213303

**Published:** 2026-10-26

**Authors:** Jun Zhang, Xi Hao, Minghui Yu, Man Zhang, Mengya Liu, Xiaodong Dai, Xiuwang Zang

**Affiliations:** 1 Peanut Research Institute, Henan Academy of Agricultural Sciences, Zhengzhou 450002, China; 2 Xinyang Academy of Agricultural Sciences, Xinyang 464000, China; 3 Henan Institute of Crop Molecular Breeding, Henan Academy of Agricultural Sciences, Zhengzhou 450002, China

**Keywords:** *Arachis hypogaea*, nitrate limitation, root spatial allocation, nitrogen accumulation, genotypic variation, nodulation, rhizosphere enzymes

## Abstract

Root system architecture can influence nitrogen (N) acquisition under low N, but genotypic variation in peanut remains poorly resolved. We evaluated 12 peanut (*Arachis hypogaea* L.) genotypes under a common low-N regime (0.15 mmol L^−1^ nitrate-N) using independent experimental modules spanning seedling, flowering–pegging, and late reproductive stages. Root morphology, TTC-reduction-based root metabolic activity, spatial biomass allocation, total plant N accumulation, nodulation, reproductive traits, and rhizosphere N-cycling enzyme activities were quantified. Yuhua 37 (YH37) showed concordant favorable performance across independent assays, ranking among the highest genotypes for N accumulation and combining long roots, high root biomass, a high root-to-shoot ratio, and high root metabolic activity. Across genotypes, seedling total N accumulation was positively correlated with total root length (Spearman’s ρ = 0.71, *p* = 0.0128) and below-9 cm root allocation (ρ = 0.68, *p* = 0.017); both remained significant after Benjamini–Hochberg adjustment (q = 0.034). Root dry weight and TTC-reduction-based root metabolic activity were positively but not significantly associated with total N accumulation. In the independent reproductive-stage assay, YH37 also showed high nodule, peg, and pod numbers and high rhizosphere urease and alkaline protease activities. Because mineral-derived and biologically fixed N were not partitioned, total plant N accumulation should not be interpreted as nitrate uptake alone. These results identify total root length and relative vertical root deployment as candidate phenotypic axes associated with total plant N accumulation under the tested low-N condition, while validation across N levels and field environments is still required.

## 1. Introduction

Nitrogen (N) is required in larger quantities than any other mineral nutrient and exerts strong control over plant growth, carbon assimilation, and crop productivity. Modern crop production has therefore relied heavily on mineral N inputs, but inefficient recovery of applied N carries substantial economic and environmental costs. Improving the ability of crops to acquire and use N at lower external supply is consequently a major objective for sustainable agriculture and crop improvement [1,2,3,4]. At the plant level, N acquisition is not determined solely by transporter capacity or tissue N metabolism. It is also constrained by the size, spatial deployment, construction cost, and physiological activity of the root system, which together determine how effectively a plant encounters and exploits temporally and spatially heterogeneous N resources [5,6,7,8].

Root system architecture (RSA) is particularly important for mobile nutrients such as nitrate. Root length, branching, depth, and the three-dimensional distribution of roots among soil domains determine the volume of soil explored and the degree of overlap among neighboring roots [9,10,11]. Nitrate itself is also a developmental signal that modifies primary and lateral root growth, allowing plants to adjust foraging behavior to local and systemic N status [8,12,13]. Work in maize and other crops has shown that root phenotypes promoting deeper or more efficient soil exploration can enhance N capture under low-N conditions [14,15,16]. The broader “steep, cheap, and deep” framework likewise emphasizes root phenes that increase access to mobile resources while limiting the metabolic cost of soil exploration [17,18,19]. These concepts imply that no single aggregate trait is expected to explain N capture across genotypes: similar root length can arise through contrasting combinations of biomass investment, specific root length, activity, and spatial deployment.

Peanut (*Arachis hypogaea* L.) provides an especially interesting system in which to study N nutrition because it combines soil mineral N uptake with symbiotic biological N fixation. Nodulation and N_2_ fixation are sensitive to external N supply, and excessive mineral N can suppress nodule formation and nitrogenase activity [20,21,22]. Isotopic studies in peanut further show that the relative contribution of soil N, fertilizer N, and nodule-derived N changes across growth stages and depends on the chemical form of supplied N [23,24]. Peanut studies have also shown that N deficiency can inhibit root growth, root vitality, nitrate assimilation, and nodulation, whereas N management alters root morphology, root distribution, biological N fixation, and N-use efficiency [25,26,27,28,29,30]. Similar work in legumes highlights RSA remodeling and root–microbe coordination as important components of N acquisition under nutrient limitation [31]. However, most peanut studies have focused on responses of one or a few cultivars to contrasting N treatments, and comparatively few studies have jointly evaluated root system size, biomass investment, physiological activity, spatial deployment, and total plant N accumulation across a broader cultivated panel. Moreover, because total plant N accumulation integrates N acquired from mineral sources and symbiotic fixation, it cannot be interpreted as equivalent to nitrate acquisition when N sources are not partitioned.

A second gap concerns root spatial deployment. Total root length and root dry weight are convenient integrative metrics, but they do not indicate where roots are positioned in three-dimensional soil space. For a mobile resource such as nitrate, allocation of roots to lower soil domains can be particularly important, whereas radial deployment determines lateral exploration around the taproot and can complement vertical deployment by increasing the volume of substrate explored [10,19]. In peanut, deeper root length has been associated with improved yield and N-use efficiency under water and N limitation [28], but comparative information on vertical and radial root biomass allocation among genotypes remains limited. Resolving these complementary spatial dimensions may help distinguish genotypes that merely produce more roots from those that deploy roots differently within the explored soil profile.

The objective of this study was to characterize genotypic variation in root system size, biomass investment, TTC-reduction-based metabolic activity, vertical and radial deployment, and total plant N accumulation across 12 cultivated peanut genotypes under a common low-N condition. The major trait groups were assessed in independent developmental modules so that genotype-level concordance could be evaluated without treating measurements from different plants or stages as longitudinal observations. We hypothesized that genotypes with greater soil exploration—particularly longer roots and greater allocation below the upper root-box layer—would show greater total plant N accumulation than genotypes characterized primarily by high root biomass alone. We also examined whether genotypes with favorable seedling root–N profiles showed concordant nodulation, reproductive, and rhizosphere traits in an independent reproductive-stage assay. Because only one low-N regime was evaluated, the study compares genotypic performance under this defined condition and does not test genotype × N interaction or establish low-N-specific tolerance.

## 2. Results

### 2.1. Peanut Genotypes Differ Substantially in Growth and Total Plant Nitrogen Accumulation Under Low N

**Figure 1:**
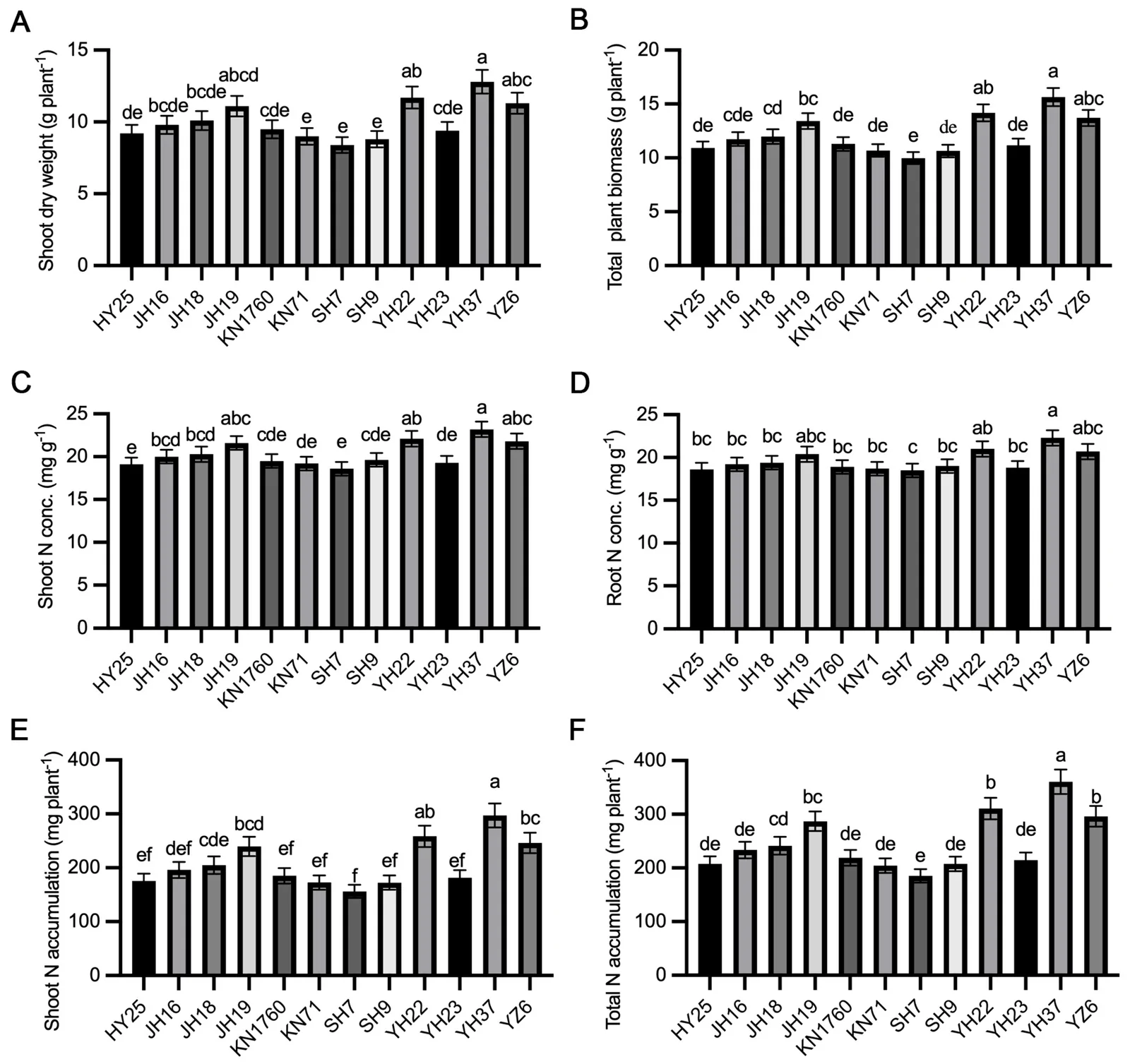
Genotypic variation in plant growth, tissue nitrogen status, and nitrogen accumulation among 12 peanut genotypes under low-nitrogen conditions. (**A**) Shoot dry weight, (**B**) total plant biomass, (**C**) shoot nitrogen (**N**) concentration, (**D**) root N concentration, (**E**) shoot N accumulation, and (**F**) total plant N accumulation. Bars represent means ± SD (*n* = 3 biological replicates). Different lowercase letters above the bars indicate significant differences among genotypes based on analysis of variance (ANOVA) with container as a blocking factor, followed by Tukey’s honestly significant difference (HSD) test (*p* < 0.05).

In the independent later-stage N-accumulation assay, low N revealed pronounced genotypic variation in plant growth and N accumulation (Figure 1). Shoot dry weight ranged from approximately 8.4 to 12.8 g plant^−1^, and total plant biomass ranged from approximately 10.0 to 15.7 g plant^−1^. YH37 had the highest mean shoot dry weight and total biomass, with YH22, YZ6, and JH19 also in the upper part of the genotype distribution. Variation in tissue N concentration was narrower than variation in biomass: shoot N concentration ranged from approximately 18.6 to 23.2 mg g^−1^ and root N concentration from approximately 18.5 to 22.3 mg g^−1^. Nevertheless, the combination of biomass production and tissue N concentration produced marked divergence in N accumulation. YH37 had the greatest shoot N accumulation (about 297 mg N plant^−1^) and total plant N accumulation (about 361 mg N plant^−1^), followed by YH22 and YZ6. The lowest total N accumulation occurred in SH7 (about 186 mg N plant^−1^), giving an approximately 1.9-fold range across the panel. Thus, genotypic differences in later-stage total plant N accumulation reflected integrated differences in biomass production and tissue N status rather than large differences in N concentration alone.

### 2.2. Genotypes Exhibit Contrasting Combinations of Root Size, Biomass Investment, Efficiency, and Metabolic Activity

**Figure 2:**
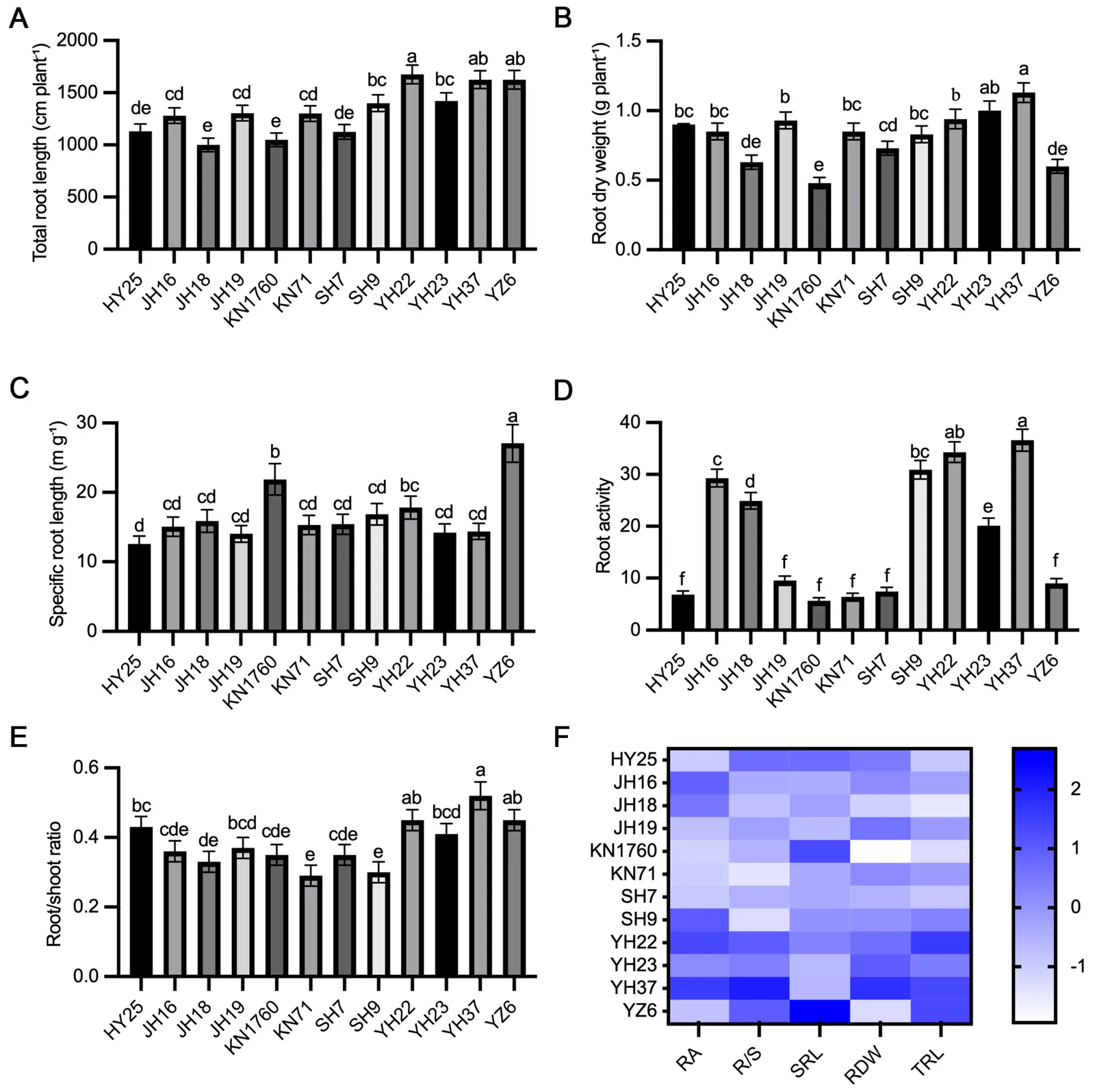
Genotypic variation in root morphological traits and TTC-reduction-based metabolic activity among 12 peanut genotypes under low-nitrogen conditions. (**A**) Total root length, (**B**) root dry weight, (**C**) specific root length, (**D**) TTC-reduction-based root metabolic activity (OD485 g^−1^ FW), and (**E**) root-to-shoot ratio. (**F**) Heatmap of Z-score-standardized mean values for the five root traits across genotypes. Bars represent means ± SD (*n* = 3 biological replicates). Different lowercase letters indicate significant differences among genotypes according to ANOVA with container as a blocking factor, followed by Tukey’s HSD test (*p* < 0.05). Positive and negative Z-scores indicate trait values above and below the across-genotype mean, respectively. RA, TTC-reduction-based root metabolic activity; R/S, root-to-shoot ratio; SRL, specific root length; RDW, root dry weight; TRL, total root length.

The seedling root screen revealed distinct root strategies among the 12 genotypes (Figure 2). Total root length varied from approximately 1000 cm plant^−1^ in JH18 to 1675 cm plant^−1^ in YH22. YH37 and YZ6 also produced long root systems (both approximately 1625 cm plant^−1^). Root dry weight showed a different ranking: YH37 had the highest mean root dry weight (1.13 g plant^−1^), followed by YH23, YH22, and JH19, whereas KN1760 and YZ6 invested comparatively little dry mass in roots. Consequently, SRL differed strongly among genotypes. YZ6 had the highest SRL (approximately 27.1 m g^−1^), and KN1760 was also high, whereas YH37 had a moderate SRL despite its large total root length. TTC-reduction-based root metabolic activity varied nearly sevenfold, from about 5.6–9.5 OD485 g^−1^ FW in the lowest-activity group to 36.6 OD485 g^−1^ FW in YH37. YH22, SH9, and JH16 also showed high metabolic activity. YH37 had the largest root-to-shoot ratio (0.52), while YH22 and YZ6 were also characterized by comparatively high belowground allocation. The standardized trait heatmap therefore identified YH37 as a genotype combining high values for several root dimensions, whereas other genotypes displayed more trait-specific strategies.

### 2.3. Vertical and Radial Root Biomass Allocation Varies Strongly Among Genotypes

**Figure 3:**
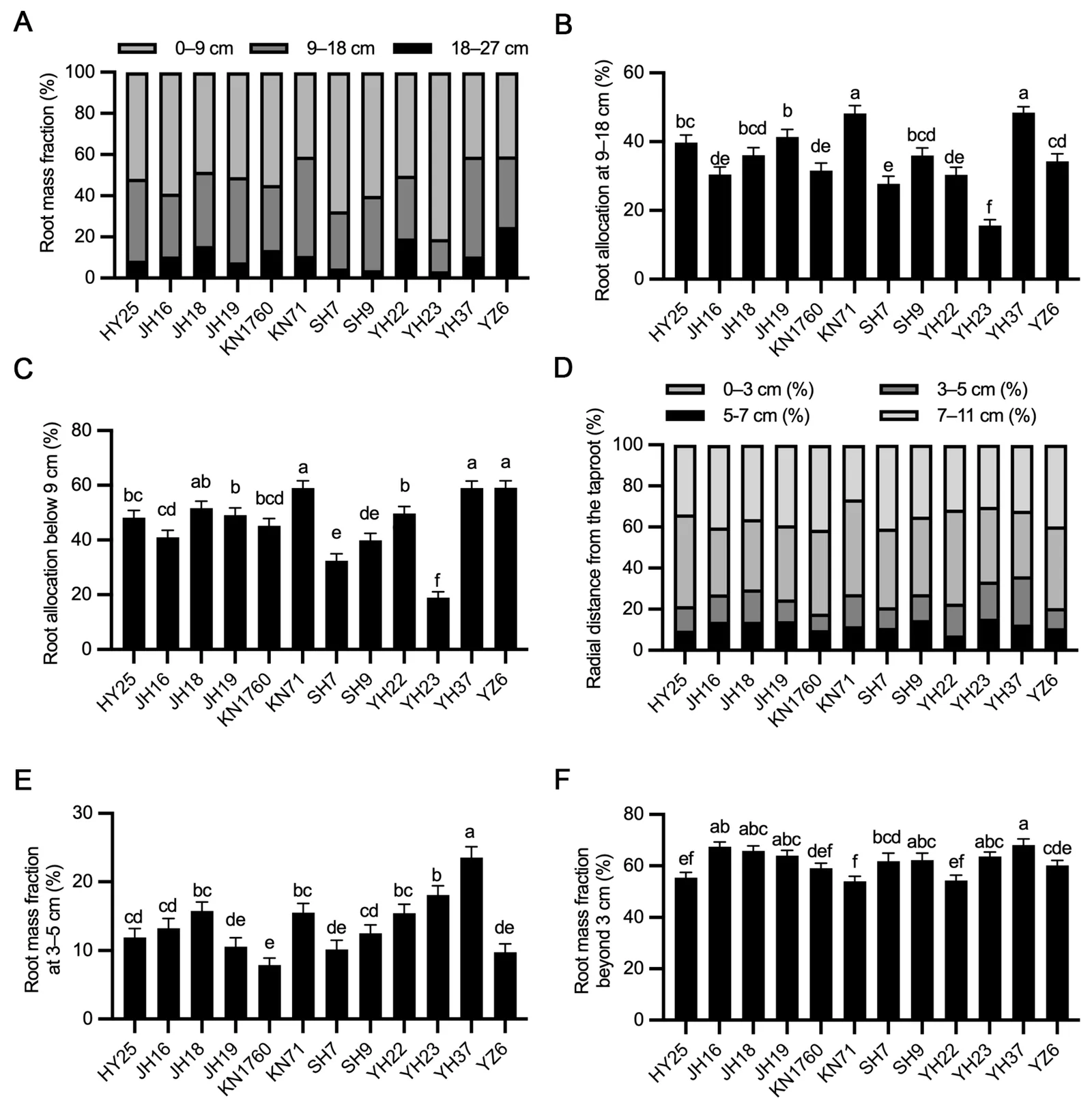
Genotypic variation in vertical and radial root biomass allocation among peanut genotypes under low-nitrogen conditions. (**A**) Vertical distribution of root mass among the 0–9, 9–18, and 18–27 cm depth intervals within the root box. (**B**) Root mass fraction allocated to the 9–18 cm depth interval (%). (**C**) Root mass fraction allocated below 9 cm within the root box (%), calculated as the combined proportion of roots in the 9–18 and 18–27 cm intervals. (**D**) Radial distribution of root mass within the 0–3, 3–5, 5–7, and 7–11 cm zones from the taproot axis. (**E**) Root mass fraction allocated to the 3–5 cm radial zone (%). (**F**) Root mass fraction distributed beyond 3 cm from the taproot axis (%), calculated as the combined proportion of roots in the 3–5, 5–7, and 7–11 cm zones. Stacked bars in (**A**,**D**) represent proportional root-mass distributions and sum to 100%. Bars in (**B**,**C**,**E**,**F**) represent means ± SD (*n* = 3 biological replicates). Different lowercase letters indicate significant differences among genotypes according to one-way ANOVA followed by Tukey’s HSD test (*p* < 0.05).

Genotypic differences were also evident in vertical root biomass deployment in the seedling root-box assay (Figure 3). YH23 concentrated approximately 81.1% of its root biomass in the 0–9 cm layer, whereas only about 41.0% of YH37 root biomass occurred in this upper layer. YH37 allocated 48.4% of its root biomass to the 9–18 cm layer, placing it in the highest statistical group together with KN71. When the 9–18 and 18–27 cm layers were combined, approximately 59.0% of YH37 root biomass occurred below 9 cm. KN71 and YZ6 showed similarly high below-9 cm allocation, but their vertical profiles differed: YZ6 had the largest fraction in the 18–27 cm layer (24.8%), whereas YH37 was characterized primarily by strong allocation to the intermediate 9–18 cm layer.

Radial allocation further differentiated the genotypes. YH37 placed only 31.9% of its root biomass within 0–3 cm of the taproot axis, while 23.5% occurred in the 3–5 cm zone—the largest 3–5 cm fraction in the panel. Its cumulative root mass fraction beyond 3 cm was approximately 68.1%. By contrast, KN71 and YH22 allocated comparatively large fractions close to the taproot axis. Thus, YH37 combined substantial allocation below the uppermost root-box layer with radial extension, producing a spatial phenotype distinct from genotypes that were strongly top-layer concentrated (e.g., YH23) or that combined high below-9 cm allocation with relatively low root biomass/activity (e.g., YZ6).

### 2.4. Root Length and Below-9 cm Root Allocation Show the Strongest Genotype-Level Associations with Total Plant Nitrogen Accumulation

**Figure 4:**
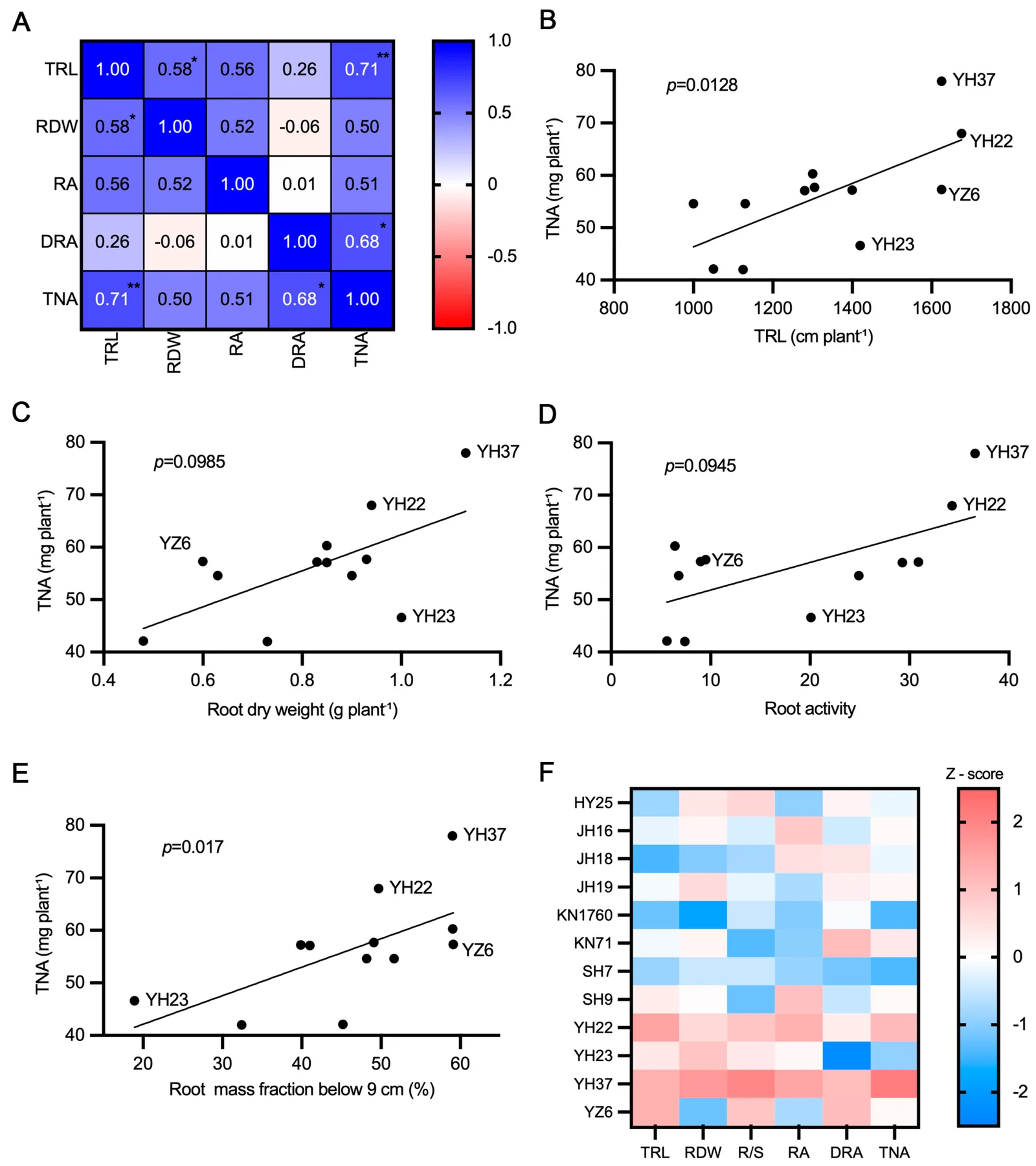
Relationships between root traits and total plant nitrogen accumulation among peanut genotypes under low-nitrogen conditions. (**A**) Spearman’s rank correlation matrix among total root length (TRL), root dry weight (RDW), TTC-reduction-based root metabolic activity (RA), below-9 cm root allocation (DRA), and total plant nitrogen accumulation (TNA). Values indicate Spearman’s ρ; * *p* < 0.05 and ** *p* < 0.01. (**B**–**E**) Relationships between TNA and TRL (**B**), RDW (**C**), RA (**D**), and DRA (**E**). Each point represents one genotype mean (*n* = 12 genotypes); each genotype mean was calculated from three biological replicate units within the corresponding assay. Solid lines are shown only to visualize overall trends; nominal *p*-values were obtained from two-tailed Spearman’s rank correlation tests. For the four prespecified root–N relationships, Benjamini–Hochberg-adjusted q-values are reported in the Results. (**F**) Heatmap of Z-score-standardized root traits and TNA across genotypes. Positive and negative Z-scores indicate values above and below the across-genotype mean, respectively. DRA is an experiment-specific metric representing the proportion of root biomass below 9 cm within the 27 cm root-box profile and was measured in a companion assay; therefore, its association with TNA is interpreted at the genotype-mean level across independent assays. R/S, root-to-shoot ratio.

Within the seedling root-screening framework, correlation analysis showed that different root dimensions were not equally associated with total plant N accumulation (Figure 4). Total root length was positively correlated with seedling total N accumulation (Spearman’s ρ = 0.71, *p* = 0.0128), and below-9 cm root allocation was also positively correlated with total N accumulation (ρ = 0.68, *p* = 0.017). Both relationships remained significant after Benjamini–Hochberg correction across the four prespecified root–N tests (q = 0.034 for each). In contrast, root dry weight (ρ = 0.50, *p* = 0.0985, q = 0.0985) and TTC-reduction-based root metabolic activity (ρ = 0.51, *p* = 0.0945, q = 0.0985) showed positive but non-significant relationships. Because below-9 cm allocation was measured in the companion root-box assay whereas total plant N accumulation was measured in the solution-culture assay, the DRA–TNA relationship represents a genotype-level association between means obtained from independent assays rather than a within-plant physiological relationship. A leave-one-genotype-out sensitivity analysis supported the robustness of the two principal associations: Spearman’s ρ remained positive at 0.63–0.84 for total root length versus total N accumulation and at 0.61–0.75 for below-9 cm allocation versus total N accumulation. Total root length was moderately associated with root dry weight and metabolic activity, whereas below-9 cm allocation was nearly independent of root dry weight and metabolic activity, indicating that relative vertical deployment captured a distinct dimension of the root phenotype. The standardized root–N heatmap emphasized this complementarity: YH37 combined positive standardized values for total root length, root dry weight, root-to-shoot ratio, metabolic activity, below-9 cm allocation, and total N accumulation; YZ6 combined high root length and below-9 cm allocation with relatively low root dry weight and metabolic activity; and YH23 combined relatively high root biomass with very low below-9 cm allocation and lower N accumulation.

### 2.5. Genotypic Variation in Nodulation, Reproductive Traits, and Rhizosphere N-Cycling Enzyme Activities

**Figure 5:**
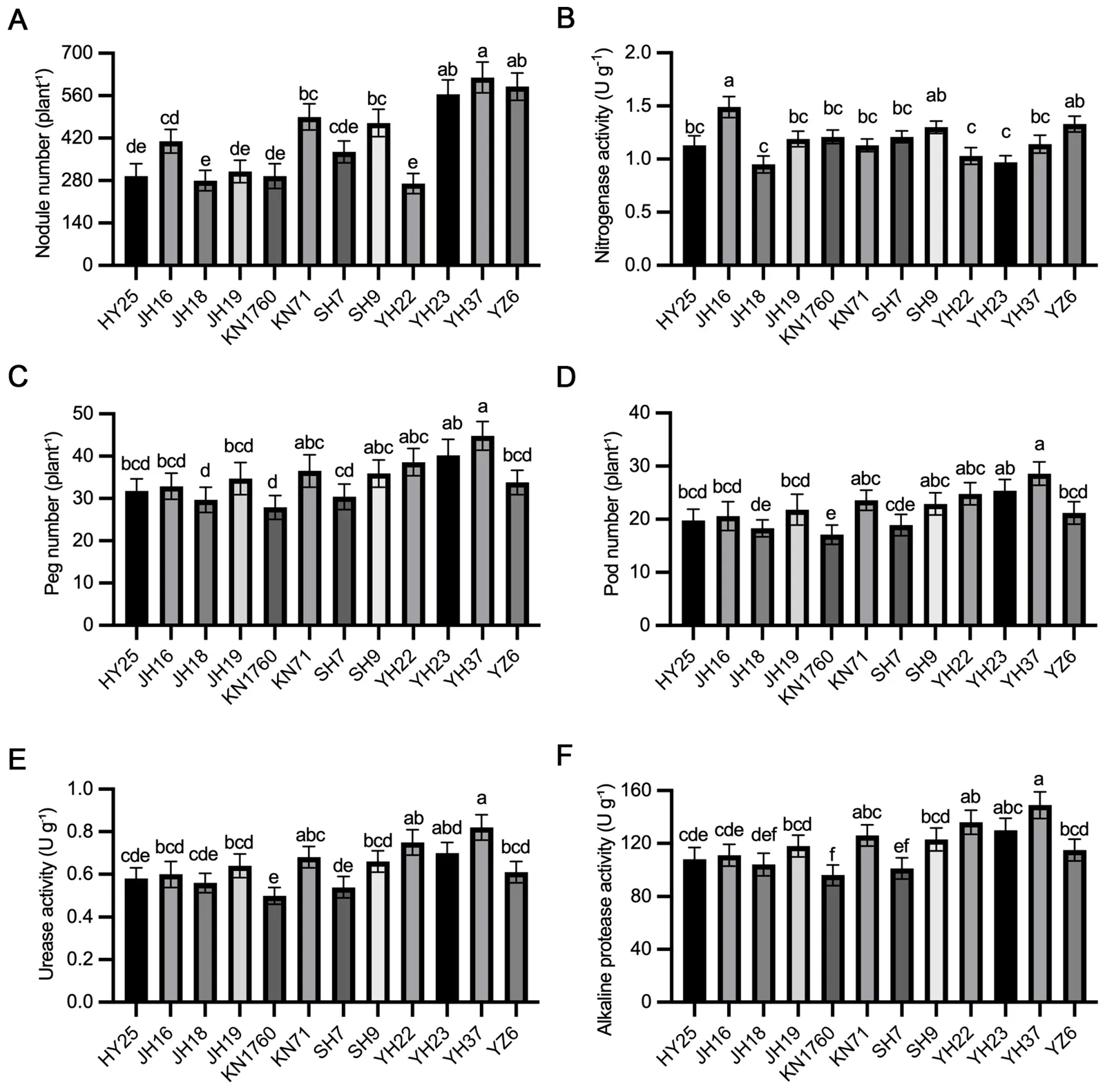
Genotypic variation in nodulation, reproductive traits, and rhizosphere N-cycling enzyme activities of peanut under low-nitrogen conditions. (**A**) Nodule number, (**B**) nitrogenase activity (U g^−1^ nodule fresh weight), (**C**) peg number, (**D**) pod number, (**E**) rhizosphere soil urease activity (U g^−1^ dry soil), and (**F**) rhizosphere soil alkaline protease activity (U g^−1^ dry soil). Bars represent means ± SD (*n* = 3 biological replicates). Different lowercase letters indicate significant differences among genotypes according to ANOVA with container as a blocking factor, followed by Tukey’s HSD test (*p* < 0.05).

In an independent whole-season low-N assay, genotypes also differed in nodulation and reproductive development (Figure 5). YH37 had the highest mean nodule number (approximately 620 nodules plant^−1^) and belonged to the highest statistical group together with YZ6 and YH23. Nitrogenase activity, however, did not follow nodule number: JH16 showed the highest mean activity (approximately 1.49 U g^−1^), while YH37 was intermediate (approximately 1.14 U g^−1^). Thus, greater nodule abundance in YH37 was not accompanied by the highest activity per unit assayed material.

YH37 also had the highest peg number (approximately 44.8 plant^−1^) and pod number (approximately 28.6 plant^−1^), with YH23 and YH22 among the next highest genotypes. Rhizosphere urease and alkaline protease activities were likewise high in YH37 relative to the panel. Because these traits were measured in a separate experiment from the seedling correlation analysis and later-stage N-accumulation assay, they are best interpreted as concordant genotype-level responses rather than as mechanistic mediators of the N-accumulation differences observed in the other modules.

## 3. Discussion

### 3.1. Genotypic Variation in Total Plant Nitrogen Accumulation Highlights Potential Root Phenotyping Targets in Peanut

The central result of this study is that cultivated peanut genotypes differ substantially in total plant N accumulation under a common low-N environment and that these differences are accompanied by contrasting root phenotypes across independent developmental modules. YH37 ranked near the upper end of N accumulation in both the seedling association dataset and the independent later-stage assay, whereas genotypes such as SH7 and KN1760 were lower in the later-stage comparison. Genetic variation in N uptake and utilization is a prerequisite for breeding crops that sustain productivity with lower N inputs [1,4]. Root-based approaches are especially attractive because natural variation in root morphology, uptake capacity, and spatial deployment can contribute to N acquisition efficiency [2,7,19]. In peanut, recent studies have emphasized strong physiological, transcriptomic, and metabolic responses of roots to N deficiency [25,26,27,30], but much of this work has focused on stress responses within one or a few cultivars. Our multi-genotype comparison extends that framework by showing that root traits relevant to performance in a low-N environment vary markedly within cultivated germplasm and are associated, at the genotype level, with total plant N accumulation.

The relatively modest genotypic range in tissue N concentration compared with the much larger range in later-stage total N accumulation is also informative. N accumulation integrates tissue N status with the capacity to produce biomass under limited N. In the later-stage assay, YH37 combined relatively high tissue N concentration with high biomass, resulting in high whole-plant N accumulation. This pattern is consistent with the broader concept that improved N performance requires coordination of N capture, assimilation, and growth rather than maximization of a single concentration-based trait [3]. It also argues for caution when using tissue N concentration alone to classify genotypes, because a high concentration in a small biomass does not necessarily represent high total plant N accumulation. Because mineral-derived and biologically fixed N were not partitioned in this study, these accumulation values should not be interpreted as direct estimates of nitrate acquisition.

### 3.2. YH37 Combines Root Investment, Metabolic Activity, and Soil Exploration, but Alternative Root Strategies Are Evident

YH37 was distinguished at the seedling stage not by a single exceptional root trait but by a combination of high root biomass, high root-to-shoot ratio, long roots, and high TTC-reduction-based root metabolic activity. This coordinated phenotype is consistent with a relatively high level of belowground investment and spatial exploration under N limitation, but TTC reduction should be interpreted as an indicator of root metabolic/dehydrogenase activity rather than a direct measurement of nutrient uptake. Peanut roots are known to respond strongly to N supply; N deficiency can suppress root growth, vitality, and nitrate-assimilation processes [25,26], and combined N and physical soil stress also causes major changes in peanut root growth, nodulation, and metabolism [27]. More broadly, legume RSA remodeling integrates nutrient signaling with root–microbe interactions and is increasingly viewed as a target for improving N acquisition [31]. The present data show that the magnitude and combination of these root traits are strongly genotype-dependent under a common low-N environment.

At the same time, the panel revealed alternative root-economic strategies. YZ6 achieved a total root length comparable with YH37 but had much lower root dry weight and TTC-reduction-based root metabolic activity, resulting in the highest SRL in the panel. High SRL is generally associated with greater root length constructed per unit biomass, a potentially less costly exploratory strategy. Conversely, YH23 invested heavily in root biomass but concentrated a large fraction of its root system in the upper root-box layer and accumulated less N in the seedling root–N association dataset. These contrasts support the view that root construction cost, length production, metabolic activity, and spatial placement are partially independent dimensions of resource acquisition [10,17,18,19]. Therefore, greater root biomass alone is unlikely to be an adequate selection criterion, while SRL alone may overlook physiological and spatial attributes that influence N capture.

### 3.3. Root Length and Vertical Deployment Are More Consistently Associated with Total Plant Nitrogen Accumulation than Root Biomass Alone

Among the root traits evaluated, total root length and below-9 cm root allocation showed the clearest genotype-level associations with seedling total N accumulation, and both associations remained significant after false-discovery-rate correction. The association involving below-9 cm allocation is specifically a cross-assay relationship between genotype means because spatial allocation and total N accumulation were measured on different plants in companion seedling experiments. Greater vertical deployment could plausibly improve access to mobile nitrate, but depth-specific nitrate depletion or uptake was not measured here; this explanation therefore remains a physiological hypothesis rather than a demonstrated mechanism. Developmental and signaling studies show that plants remodel RSA in response to nitrate availability and internal N status [6,8,12,13]. Root-foraging theory and empirical studies in maize likewise show that longer, sparser, or vertically extensive root systems can increase nitrate capture and performance under low N [14,15,16,19]. In peanut, Ding et al. [28] reported positive relationships among deeper root length, yield, and N-use efficiency under combined water and N treatments. Our data are consistent with this broader principle, while the 27 cm root-box profile means that the present below-9 cm metric should be interpreted as relative vertical deployment within the phenotyping system rather than field-scale deep rooting.

The spatial data also show why a simple shallow-versus-deep classification can be insufficient. YH37, KN71, and YZ6 all allocated approximately 59% of their root biomass below 9 cm, but they differed sharply in root biomass, metabolic activity, and N accumulation. YZ6 had the largest fraction in the 18–27 cm layer and high SRL, whereas YH37 combined strong 9–18 cm allocation with high root biomass and metabolic activity. The weak correlations between below-9 cm allocation and either root dry weight or root metabolic activity further indicate that spatial deployment represents an axis of root phenotype that is not captured by biomass or metabolic activity alone. This complementarity is consistent with RSA theory, in which the utility of a root system emerges from combinations of phenes rather than a single dimension [8,9,18].

Root dry weight and TTC-reduction-based root metabolic activity were positively, but not significantly, associated with total N accumulation across the 12 genotype means. The TRL–TNA association remained positive in leave-one-genotype-out analysis (ρ = 0.63–0.84), indicating that its direction was not determined by a single genotype. Nevertheless, this analysis does not control for whole-plant biomass; genotypes with greater overall growth may simultaneously develop longer roots and accumulate more N. We therefore interpret the association as genotype-level covariation rather than evidence of an independent causal effect of root length. Root biomass and metabolic activity differentiated genotypes and contributed to the distinctive YH37 phenotype, but the strongest statistical evidence for root–N associations was obtained for total root length and relative vertical deployment.

### 3.4. Nodulation and Rhizosphere Traits Complement, but Do Not Fully Explain, the High-N-Accumulation Phenotype

As a legume, peanut can acquire N both from the soil and through symbiotic N_2_ fixation, so root architectural traits interact with a second N-acquisition pathway mediated by nodules. Symbiotic function requires coordination of nodule development, carbon supply, metabolite transport, and rhizobial nitrogenase activity [21,22]. External mineral N can suppress nodulation and N_2_ fixation, and the relative contributions of fertilizer/soil N and fixation vary with N supply in legumes [20,32]. Isotope-based work in peanut further demonstrates that the contributions of soil N, fertilizer N, and nodule-derived N vary with N form and crop developmental stage [23,24], while optimization of early N supply can improve peanut biological N fixation [29]. In our reproductive-stage assay, no defined Bradyrhizobial inoculant was applied, and the commercially purchased substrate was not sterilized; nodulation therefore depended on compatible indigenous rhizobia present in the substrate. Consequently, cultivar differences in nodulation may partly reflect genotype × resident-rhizobial-community compatibility and should not be interpreted as intrinsic symbiotic capacity alone.

YH37 had a high nodule number but did not have the highest nitrogenase activity, providing an important counterpoint: nodule abundance and activity per unit assayed material are not interchangeable. JH16 exhibited the highest nitrogenase activity, whereas YH37 was intermediate. Similar complexity has been reported in studies that partition soil N uptake and nodule-derived N in peanut, where both root morphology and nodule properties contribute to whole-plant N nutrition [24]. Without isotopic partitioning or whole-plant estimates of biological N fixation, the present data cannot determine how much of the genotype differences in total N accumulation arose from symbiotic fixation versus mineral N uptake. The results therefore support phenotype-level associations rather than mechanistic attribution to N_2_ fixation. Likewise, the higher peg and pod numbers observed for YH37 should be interpreted as reproductive development indicators rather than as final yield performance, because seed yield, pod weight, and harvest index were not measured.

Rhizosphere urease and alkaline protease activities were also high in YH37 in the reproductive-stage assay. These enzymes participate in transformations of organic and urea-derived N pools and can reflect microbial and substrate processes that influence N availability. Nevertheless, extracellular enzyme activity is controlled by multiple biological and physicochemical factors [33], and the current measurements should not be interpreted as evidence that enzyme activity caused greater plant N uptake. Instead, they indicate that YH37 was associated with relatively high rhizosphere N-cycling potential in a separate experimental module.

### 3.5. Implications, Limitations, and Future Directions

The results identify total root length and relative vertical deployment as candidate phenotypic axes associated with plant N accumulation under the tested low-N condition, while the broader YH37 phenotype suggests that biomass allocation and metabolic activity may complement architecture. These traits are measurable in controlled phenotyping systems, and the pronounced contrasts among YH37, YH22, YZ6, and YH23 provide potentially useful parental combinations for mapping populations, trait-dissection studies, and future investigations of the genetic basis of root architecture and plant N accumulation. However, their value as breeding targets will depend on repeatability across contrasting N supplies, soils, developmental stages, and field environments.

Several limitations should temper interpretation. First, the root–N associations were based on genotype means for only 12 cultivars and therefore establish genotype-level correlation rather than causation or plant-level prediction; the leave-one-genotype-out analysis nevertheless indicated that the two principal associations were not dependent on any single cultivar. Second, the study presents genotypic comparisons within one low-N regime; without parallel inference across an adequate-N treatment, the results cannot distinguish low-N-specific adaptation from general vigor or quantify genotype × N interaction. Third, the phenotyping modules were independent experiments conducted at different developmental stages, so cross-module concordance should be interpreted as convergence at the genotype level rather than as longitudinal responses of the same plants. Fourth, solution culture, controlled container culture, and the 27 cm root boxes simplify soil heterogeneity; in addition, the 4 cm thickness of the root boxes physically constrained three-dimensional root expansion, so the observed spatial patterns represent relative allocation within the phenotyping system rather than unrestricted field root architecture. Fifth, N sources were not partitioned using 15 N or another direct method, so mineral N uptake and biological N fixation could not be separated. Sixth, because no defined rhizobial inoculant was applied and the substrate was non-sterilized, genotype differences in nodulation may partly reflect compatibility with resident rhizobia. Finally, rhizosphere enzyme activities are integrative indicators and do not identify the microbial taxa or substrates responsible for N cycling. Future work should therefore combine multi-N-level field validation, repeated environments, isotopic N tracing, defined rhizobial treatments where appropriate, and genetic analysis of contrasting genotypes to test whether the root architectural traits identified here are stable selection targets for improved N nutrition.

## 4. Materials and Methods

### 4.1. Plant Materials and Experimental Framework

Twelve cultivated peanut genotypes were evaluated: Huayu 25 (HY25), Jihua 16 (JH16), Jihua 18 (JH18), Jihua 19 (JH19), Kainong 1760 (KN1760), Kainong 71 (KN71), Shanhua 7 (SH7), Shanhua 9 (SH9), Yuhua 22 (YH22), Yuhua 23 (YH23), Yuhua 37 (YH37), and Yuanzha 6 (YZ6). The 12 cultivars were selected to form an agronomically relevant cultivated panel spanning several peanut cultivar series (Huayu, Jihua, Kainong, Shanhua, Yuhua, and Yuanzha) and to capture contrasting agronomic phenotypes for comparative phenotyping. The purpose was to sample useful phenotypic diversity among cultivated peanut materials rather than to claim exhaustive representation of peanut genetic diversity. Full cultivar names, abbreviations, and Chinese cultivar names are provided in Supplementary Table S1. Experiments were conducted in controlled-environment facilities at the Peanut Research Institute, Henan Academy of Agricultural Sciences, Zhengzhou, Henan, China. Plants were maintained under a 14 h light/10 h dark photoperiod, day/night temperatures of 30 ± 2/25 ± 2 °C, and relative humidity of approximately 60–70%.

All genotype comparisons reported in this study were conducted under a single common low-N regime. The low-N nutrient solution supplied 0.15 mmol L^−1^ total N exclusively as nitrate, provided by 0.0177 g L^−1^ Ca(NO_3_)_2_·4H_2_O (equivalent to 0.075 mmol L-1 Ca(NO_3_)_2_). The 0.15 mmol L^−1^ N level was used as an operational low-N screening concentration for comparative peanut phenotyping rather than as a universal physiological threshold. This choice is consistent with Zhang et al. [34], who screened 127 peanut germplasm resources and identified 0.15 mM N for 24 d as an appropriate low-N hydroponic screening condition. The basal solution also contained 0.12 g L^−1^ MgSO_4_·7H_2_O, 0.10 g L^−1^ CaCl_2_·2H_2_O, 0.10 g L^−1^ KH_2_PO_4_, 0.15 g L^−1^ Na_2_HPO_4_·12H_2_O, and 0.005 g L^−1^ C_6_H_5_O_7_Fe·5H_2_O. CaCl_2_·2H_2_O was included to provide Ca without introducing additional N. A micronutrient stock solution was added at 1 mL L^−1^ of final nutrient solution; per liter of stock, it contained 0.02 g H_2_MoO_4_, 1.81 g MnSO_4_, 2.86 g H_3_BO_3_, 0.80 g CuSO_4_·5H_2_O, and 0.22 g ZnSO_4_·7H_2_O. The nutrient-solution pH was adjusted to 5.8–6.0 with HCl or NaOH. The same low-N nutrient formulation was used in all three experimental modules; in container-culture and root-box experiments, the solution was supplied to the substrate rather than used as a hydroponic bath. All chemical reagents used for nutrient solution preparation were purchased from Beyotime Biotechnology (Shanghai, China). All analyses therefore represent genotypic comparisons within a single low-N environment rather than a genotype × N interaction.

The study comprised three complementary phenotyping modules conducted at different developmental stages rather than a single destructive harvest. First, seedling-stage root screening was conducted approximately 30 days after emergence (DAE). For the solution-culture assay, three separately maintained rectangular plastic boxes (external dimensions: 60 × 40 × 20 cm; length × width × height) were fitted with 96-hole lids (12 rows × 8 holes; approximately 5 cm between adjacent planting holes), and each box contained 35 L of nutrient solution. All 12 genotypes were represented in each box, with eight seedlings of one genotype occupying each row. The genotype row order differed among the three boxes. During cultivation, the roots extended naturally downward into the nutrient solution, and no visible intermingling or entanglement among neighboring root systems was observed. At every solution renewal (every other day), a fresh common batch of nutrient solution was prepared in a large reservoir, adjusted to pH 5.8–6.0, and distributed to the three boxes (35 L per box); the used solution was discarded. The positions of the boxes were exchanged after each renewal to minimize fixed positional effects. Before subsequent phenotyping, the largest and smallest seedlings in each genotype row were excluded, and the remaining six seedlings were retained for the relevant measurements. Seedlings within each genotype-by-box row were treated as subsamples and their measurements were summarized to one box-specific observation per genotype (*n* = 3). The TTC assay used separately sampled root material rather than the roots used for scanning and dry-weight determination (Section 4.3). This module quantified total root length, root dry weight, specific root length, root-to-shoot ratio, TTC-reduction-based root metabolic activity, and seedling total N accumulation. A companion sand-filled root-box assay was conducted at the same stage for vertical and radial root distribution, with three independent root boxes per genotype. Genotype means from these companion seedling assays formed the root–N association dataset shown in Figure 2, Figure 3 and Figure 4.

For both later-stage container-culture modules, plants were grown in plastic turnover containers measuring 100 × 60 × 35 cm (length × width × height; nominal geometric volume approximately 210 L), each containing approximately 50 kg of commercially purchased, non-sterilized growth substrate. Initial extractable nitrate-N (NO_3_^−^-N) was approximately 25.2 mg kg^−1^ substrate, calculated from an extract concentration of 0.18 mmol N L^−1^ using a 1:10 (*w*/*v*) substrate-to-extractant ratio. The low-N nutrient solution was applied at 1 L per container every 3 d by uniform surface spraying, and excess water or nutrient solution was allowed to drain through holes in the bottom of the containers. In each later-stage assay, all 12 genotypes were represented within each of three replicate containers, with cultivar positions varied among containers; container positions were periodically exchanged to reduce positional effects. For each genotype, the genotype-by-container group was the replicate unit (*n* = 3), and plants sampled within that group were not counted as independent container replicates. Genotypes within the same container shared the substrate and irrigation environment. The second experimental module was harvested at approximately 55 DAE, corresponding to the flowering–pegging transition, and quantified whole-plant growth, tissue N concentration, and total plant N accumulation with three independent biological replicate units per genotype (Figure 1). Consequently, absolute root-biomass and total-N-accumulation values in Figure 1 are not expected to match those in Figure 2, Figure 3 and Figure 4; the TNA values used in Figure 4 were obtained exclusively from the seedling root-screening module.

Third, a whole-season low-N container-culture assay was maintained through reproductive development and sampled at approximately 95 DAE, after pod set, to evaluate nodulation, nitrogenase activity, peg and pod formation, and rhizosphere N-cycling enzyme activities. Three independent biological replicate units were used per genotype (Figure 5). The three modules used the same genotype panel but were analyzed as independent experiments; measurements from different modules were not treated as repeated observations from the same plants or pooled into a common ANOVA.

### 4.2. Biomass, Nitrogen Concentration, and Nitrogen Accumulation

At each harvest, plants were separated into shoots and roots, and roots were gently cleaned of adhering substrate. Tissues were heat-treated at 105 °C for 20 min and then oven-dried at 80 °C to constant mass before weighing, following the drying procedure used in peanut root phenotyping by Li et al. [26]. Total plant biomass was calculated as the sum of shoot and root dry mass, and the root-to-shoot ratio was calculated as root dry weight divided by shoot dry weight. Dried tissues were finely ground (<0.5 mm), and total N concentration was determined by a semi-micro Kjeldahl digestion–distillation procedure using a Kjeldahl nitrogen analyzer (Hengmei, Shandong Hengmei Electronic Technology Co., Ltd., Weifang, China), following Bremner and Mulvaney [35]. Shoot and root N concentrations were expressed on a dry-mass basis.

Shoot N accumulation was calculated as shoot dry weight multiplied by shoot N concentration, and root N accumulation was calculated analogously. Total plant N accumulation was calculated as the sum of shoot and root N accumulation. These accumulation-based variables were interpreted as measures of total plant N accumulation within each assay rather than as direct measurements of nitrate uptake, because mineral-derived and biologically fixed N were not partitioned. Because Figure 1 and Figure 4 represent independent developmental modules, their N-accumulation values were interpreted only within the corresponding experiment and were not directly compared on an absolute basis.

### 4.3. Root Morphology and TTC-Reduction-Based Metabolic Activity

For the seedling root-morphology assay, intact root systems were gently washed, spread in a shallow layer of water to minimize overlap, and scanned with an Epson Perfection V800 Photo flatbed scanner (Seiko Epson Corp., Nagano, Japan). Root images were analyzed with WinRHIZO 2007 (Regent Instruments Inc., Québec, QC, Canada) to obtain total root length, following established peanut root-phenotyping procedures [26,27]. Specific root length (SRL) was calculated as total root length divided by root dry weight and expressed as root length per unit root dry mass. After imaging, roots were dried to constant mass for root dry-weight determination.

Root physiological status was quantified by the triphenyl tetrazolium chloride (TTC) reduction method following Li et al. [25]. Because TTC reduction primarily reflects metabolic/dehydrogenase activity rather than nutrient uptake directly, the variable is referred to here as TTC-reduction-based root metabolic activity. Fresh roots were cut into approximately 1 cm segments, weighed, and immersed in 20 mL of 0.6% TTC prepared in 0.1 mol L^−1^ phosphate buffer (pH 7.4). After incubation for 24 h, roots were rinsed three times with deionized water and the formed triphenyl formazan was extracted in 20 mL of 95% ethanol at 85 °C for 10 min. Absorbance was measured at 485 nm, and activity was expressed as OD485 g^−1^ fresh weight (FW). Roots used for morphology were first scanned and then oven-dried for dry-weight determination, whereas the destructive TTC assay was performed on separate, independently sampled root material from plants allocated to the TTC measurement; root tissue used for TTC was not reused for scanning or dry-weight determination. All genotypes were processed under identical assay conditions.

### 4.4. Vertical and Radial Root Biomass Allocation

Root spatial deployment was quantified in a companion sand-filled root-box experiment conducted at the same seedling stage as the morphology/metabolic-activity screen. Each root box had internal dimensions of 27 cm high × 22 cm wide × 4 cm thick and was filled with approximately 3.5 kg of washed quartz sand (0.5–1.0 mm particle size). One seedling was positioned centrally along the upper edge of each box, and three independent root boxes were used per genotype. The common low-N nutrient solution described above was supplied regularly to maintain approximately 70% of substrate water-holding capacity. At harvest, boxes were dismantled sequentially and roots were recovered from defined spatial compartments by careful washing and sieving. Vertical zones were 0–9, 9–18, and 18–27 cm, while radial zones were 0–3, 3–5, 5–7, and 7–11 cm from the taproot axis.

Root material from each compartment was dried and weighed separately. The root mass fraction of each zone was calculated as the dry mass recovered from that zone divided by total recovered root dry mass and multiplied by 100. The metric abbreviated DRA in Figure 4 was operationally defined as below-9 cm root allocation, i.e., the combined root-mass fraction in the 9–18 and 18–27 cm layers. Root allocation beyond 3 cm was calculated as the combined fraction in the 3–5, 5–7, and 7–11 cm radial zones. Because the full experimental profile was 27 cm deep, DRA should be interpreted as relative vertical deployment within the root box rather than as field-scale deep rooting. In addition, the 4 cm thickness of the boxes physically constrained three-dimensional root expansion; thus, the measured patterns represent relative spatial allocation within this standardized phenotyping system rather than unrestricted three-dimensional root architecture in field soil. The stacked distributions in Figure 3A,D were used descriptively because their component fractions are compositional and sum to 100%; inferential comparisons focused on the prespecified derived fractions shown in Figure 3B,C,E,F.

### 4.5. Nodulation, Reproductive Traits, and Rhizosphere Enzyme Activities

In the whole-season low-N assay, no defined Bradyrhizobium inoculant was applied, and the commercially purchased growth substrate was not sterilized before planting; nodulation therefore depended on compatible indigenous rhizobia present in the substrate. Nitrogenase activity was estimated on freshly harvested nodulated root systems before washing, using the acetylene-reduction assay and the general approach established for groundnut nodules [36,37]. Fresh nodulated roots were enclosed in sealed glass vessels, and acetylene was introduced to 10% (*v*/*v*) of the headspace. Samples were incubated at 25 °C for 1 h, after which ethylene production was quantified by gas chromatography with a flame-ionization detector (FID; Agilent Technologies, Inc., Santa Clara, CA, USA). One activity unit (U) was defined operationally as 1 μmol C_2_H_4_ produced h^−1^ under the assay conditions, and activity was expressed as U g^−1^ nodule fresh weight. After the assay, root systems were gently washed, and nodules were recovered and counted on a per-plant basis. Peg number and pod number were counted for each plant during the late reproductive sampling period. These measurements were used to characterize symbiotic and reproductive performance rather than to partition the absolute sources of plant N.

Rhizosphere soil was collected as the soil remaining closely attached to roots after gentle shaking, and activities were expressed on a dry-soil-equivalent basis. Urease activity was determined by incubating soil with urea and quantifying the released ammonium colorimetrically using the phenol–hypochlorite reaction, following the principle of Kandeler and Gerber [38]. Alkaline protease activity was assayed with casein as substrate, and released tyrosine equivalents were quantified colorimetrically following established casein-based soil protease procedures [39,40]. For consistency with Figure 5, one urease unit (U) was defined as the activity releasing 1 μg NH_4_^+^-N h^−1^ under the assay conditions, whereas one alkaline-protease unit was defined as the activity releasing 1 μg tyrosine equivalent h^−1^; both activities were normalized to 1 g dry soil and reported as U g^−1^ dry soil. These extracellular enzyme activities were interpreted as indicators of rhizosphere N-transformation potential rather than as direct measures of plant N uptake [33].

### 4.6. Statistical Analysis

Data are presented as means ± standard deviation (SD). For the seedling solution-culture assay, seedlings within each genotype row were treated as subsamples and their measurements were summarized to one genotype-by-box observation, yielding three box-specific observations per genotype (*n* = 3). For the later-stage shared-container assays, measurements from plants within each genotype-by-container group were likewise summarized to one observation, with three container-level observations per genotype. For the shared-container assays (Figure 1, Figure 2 and Figure 5), genotype effects were evaluated by analysis of variance (ANOVA) with genotype as the treatment factor and container as a blocking factor to account for the shared-container environment. The companion sand-filled root-box assay (Figure 3), with three physically separate root boxes per genotype, was analyzed using one-way ANOVA. Genotype means were compared using Tukey’s honestly significant difference (HSD) test (*p* < 0.05). The stacked proportional profiles in Figure 3A,D were summarized descriptively because their component fractions sum to 100%; ANOVA was applied to the derived allocation traits in Figure 3B,C,E,F. Residual normality was assessed using normal Q–Q plots and the Shapiro–Wilk test, whereas homogeneity of variance was evaluated using residual-versus-fitted plots and Levene’s test. No substantial violations of these assumptions were detected, and no data transformations were required. Genotype-level associations in the seedling root-screening framework were assessed using two-tailed Spearman’s rank correlation based on one mean value per genotype (*n* = 12 genotypes). Four root–N relationships (total root length, root dry weight, TTC-reduction-based root metabolic activity, and below-9 cm root allocation versus total N accumulation) were specified a priori. Nominal *p* values are shown in Figure 4; these four *p* values were also adjusted using the Benjamini–Hochberg false-discovery-rate procedure. To evaluate sensitivity to influential genotypes, leave-one-genotype-out analyses were conducted for the two principal associations (TRL–TNA and DRA–TNA) by sequentially omitting each genotype and recalculating Spearman’s ρ. The full correlation matrix was interpreted as exploratory. Linear trend lines were included only to aid visualization; inference was based on Spearman’s correlation. Trait heatmaps were generated after standardizing each trait across genotypes as Z = (x − mean) / SD. Statistical analyses and graphing were performed in GraphPad Prism 10 (GraphPad Software, Boston, MA, USA).

## 5. Conclusions

The 12 peanut genotypes displayed substantial variation in total plant N accumulation and multiple dimensions of the root phenotype under a common low-N environment. YH37 ranked highly for N accumulation in independent developmental assays and combined high root investment and TTC-reduction-based metabolic activity with a distinctive spatial pattern characterized by strong allocation to the 9–18 cm layer and substantial radial extension. Within the seedling association dataset, total root length and below-9 cm root allocation were significantly associated with total plant N accumulation and remained significant after correction for the four prespecified root–N tests, whereas root dry weight and metabolic activity showed positive but weaker relationships. Independent reproductive-stage measurements further distinguished YH37 for nodulation, reproductive traits, and rhizosphere N-cycling enzyme activities, but these data do not establish a single causal mechanism. Because mineral-derived and biologically fixed N were not partitioned, total plant N accumulation should not be interpreted as nitrate acquisition alone. Overall, total root length and relative vertical root deployment represent candidate phenotypic axes associated with plant N accumulation under the tested low-N condition. Their utility as selection criteria for low-N adaptation requires validation under contrasting N supplies and field environments.

## Data Availability

The original contributions presented in this study are included in the article and Supplementary Materials. Further inquiries can be directed to the corresponding author.

## Supplementary Materials

The following supporting information can be downloaded at: https://www.mdpi.com/article/10.3390/plants15213303/s1, Table S1, Peanut genotypes used in this study; Table S2, Genotype-level mean values used in Figure 4 root-trait–total plant N accumulation analyses.
