# Supplementary material for: Genotypic Variation in Root System Architecture Is Associated with Total Plant Nitrogen Accumulation in Peanut (Arachis hypogaea L.) Under Low-Nitrogen Conditions
Source: Plants (Basel). 2026 Oct 26;15(21):3303. doi: 10.3390/plants15213303 (PMC13765587; doi:10.3390/plants15213303)
Supplement: Supplementary file 1 [file plants-15-03303-s001.zip › plants-4630097-supplementary.pdf]

Supplementary Materials

Genotypic Variation in Root System Architecture Is Associated with Total Plant Nitrogen Accumulation in Peanut (*Arachis hypogaea L.*) under Low-Nitrogen Conditions

Table S1. Peanut genotypes used in this study.

Abbreviation	Cultivar name	Chinese cultivar name
HY25	Huayu 25	花育 25
JH16	Jihua 16	冀花 16
JH18	Jihua 18	冀花 18
JH19	Jihua 19	冀花 19
KN1760	Kainong 1760	开农 1760
KN71	Kainong 71	开农 71
SH7	Shanhua 7	山花 7 号
SH9	Shanhua 9	山花 9 号
YH22	Yuhua 22	豫花 22
YH23	Yuhua 23	豫花 23
YH37	Yuhua 37	豫花 37
YZ6	Yuanzha 6	远杂 6 号

Note: All materials are cultivated peanut (*Arachis hypogaea L.*) genotypes included in the comparative panel. English cultivar names are transliterations of the Chinese cultivar names used in the experimental records. Abbreviations used in the main text and figures correspond to those listed above.

Table S2. Genotype-level mean values used in the Figure 4 root-trait–total plant N accumulation analyses.

Genotype	TRL (cm plant^{-1})	RDW (g plant^{-1})	RA (OD485 g^{-1} FW)	DRA (%)	TNA (mg N plant^{-1})
HY25	1130.00	0.90	6.80	48.19	54.60
JH16	1280.00	0.85	29.30	41.01	57.10
JH18	1000.00	0.63	24.90	51.64	54.60
JH19	1305.00	0.93	9.50	49.08	57.70
KN1760	1050.00	0.48	5.60	45.18	42.10
KN71	1300.00	0.85	6.40	59.03	60.30
SH7	1125.00	0.73	7.40	32.42	42.00
SH9	1400.00	0.83	30.90	39.89	57.20
YH22	1675.00	0.94	34.30	49.71	68.00
YH23	1420.00	1.00	20.10	18.92	46.60
YH37	1625.00	1.13	36.60	59.01	78.00
YZ6	1625.00	0.60	9.00	59.11	57.30

Note: Values are the genotype-level means entered into the Figure 4 Spearman correlation analyses. TRL, total root length; RDW, root dry weight; RA, TTC-reduction-based root metabolic activity; DRA, below-9-cm root allocation within the 27-cm root-box profile; TNA, total plant N accumulation. Each genotype mean was calculated from three biological replicate units within the corresponding assay. DRA was obtained from the companion root-box assay, whereas TRL, RDW, RA, and TNA were obtained from the seedling solution-culture assay. TNA was calculated from shoot and root dry mass and their respective tissue N concentrations as described in Section 4.2 of the main manuscript; these component variables were not entered as separate variables in the Figure 4 correlation matrix.
